# High urinary excretion rate of glucose attenuates serum uric acid level in type 2 diabetes with normal renal function

**DOI:** 10.1007/s40618-021-01513-8

**Published:** 2021-01-29

**Authors:** Y. Qin, S. Zhang, S. Cui, X. Shen, J. Wang, X. Cui, M. Zuo, Z. Gao, J. Zhang, J. Yang, H. Zhu, B. Chang

**Affiliations:** 1 NHC Key Laboratory of Hormones and Development, Tianjin Key Laboratory of Metabolic Diseases, Chu Hsien-I Memorial Hospital & Tianjin Institute of Endocrinology, Tianjin Medical University, Tianjin, China; 2 Department of Endocrinology, First Affiliated Hospital of Gannan Medical University, Ganzhou, Jiangxi China; 3 Department of Epidemiology and Biostatistics, School of Public Health, Tianjin Medical University, Tianjin, China; 4 Department of Endocrinology, Tianjin First Central Hospital, The First Center Clinical College of Tianjin Medical University, Tianjin, China

**Keywords:** Diabetes, Urinary excretion rate of glucose, Uric acid, Hyperuricaemia, Renal clearance of uric acid

## Abstract

**Aims/Introduction:**

The relationship between urinary excretion rate of glucose (UEGL) and uric acid (UA) metabolism in adults with type 2 diabetes (T2D) remains unclear to date. This study aimed to investigate the relationships of UEGL with serum UA (SUA), urinary excretion rate of uric acid (UEUA), and renal clearance of uric acid (CLUA) in adults with T2D. We hypothesised that high UEGL increases UA excretion, which in turn leads to lower SUA.

**Materials and methods:**

This was a cross-sectional study of 635 inpatients with T2D recruited between 2018 and 2019. The relationships of UEGL with UEUA, CLUA, and hyperuricaemia were assessed using analysis of covariance and multivariate regression analysis.

**Results:**

Patients in the higher quartile of UEGL tended to have lower SUA levels than those in the lower quartile. In contrast, patients in the higher quartile of UEGL tended to have higher CLUA (*p* for trend < 0.0001), and a similar trend was observed for UEUA. In adjusted multivariable linear regression model, UEGL was negatively correlated with SUA (*β* = − 0.023, 95% CI − 0.034 to − 0.013, *p* < 0.0001). However, positive correlations of UEGL with UEUA (β = 0.046, 95% CI 0.018–0.074, *p* = 0.001) and CLUA (*β* = 0.063, 95% CI 0.042–0.085, *p* < 0.0001) were found. Furthermore, consistent significant inverse associations were observed between quartiles of UEGL and hyperuricaemia in the adjusted multivariate logistic regression model.

**Conclusions:**

A high UEGL level was positively correlated with UEUA and CLUA. Moreover, it was inversely associated with SUA level, and a consistently increased UEGL level reduced the risk of hyperuricaemia in patients with T2D.

## Introduction

Elevated serum uric acid (SUA) is associated with a higher risk of hypertension, cardiovascular disease, and diabetic kidney disease in patients with type 2 diabetes (T2D) [1–3]. In this context, lowering uric acid (UA) levels could prevent the progression of complications in patients with chronic diseases. Several studies have shown that lower SUA is associated with poor glycaemic control [4–6]. Furthermore, hyperglycaemia and glucosuria due to worsening glycaemic control was found to be correlated with a decrease in SUA [4, 7]. These suggest that diabetes may affect UA metabolism. Moreover, hyperuricosuria and hypouricaemia were found in familial renal glucosuria (i.e., mutation in the gene encoding the sodium-glucose co-transporter 2 (SGLT2) protein) [8]. Increasing evidence has also shown that SUA levels are decreased in individuals with T2D following treatment with SGLT2 inhibitors, an approved oral hypoglycaemic drug [9]. The SGLT2 inhibitor-induced SUA reduction may be attributed to the increased glucosuria that boosts UA excretion in urine through alteration of UA transport activity [10]. However, the association between UA metabolism and urinary excretion rate of glucose (UEGL) in diabetes patients treated with hypoglycaemic regimens remains unclear to date. Thus, we aimed to clarify the relationships of UEGL with SUA, urinary excretion rate of uric acid (UEUA), and renal clearance of uric acid (CLUA) in adults with T2D. We hypothesised that high UEGL increases UA excretion, which in turn leads to lower SUA.

## Materials and methods

### Study design and participants

This single-centre cross-sectional study was reviewed and approved by the Medical Ethics Committee of Tianjin Medical University Chu Hsien-I Memorial Hospital (Ethics Approval Number: DXBYYhMEC2018-17) and was conducted in accordance with the tenets of the Declaration of Helsinki. All patients provided written informed consent.

We evaluated 635 patients with T2D who were admitted to Tianjin Medical University Chu Hsien-I Memorial Hospital between 2018 and 2019. T2D was diagnosed according to the World Health Organization 2009 criteria. To rule out the effects of renal insufficiency on uric acid excretion, we included the participants with estimated glomerular filtration rate (eGFR) > 90 ml/min/1.73 m^2^ in accordance with the Kidney Disease: Improving Global Outcomes guideline in 2012 [11]. Patients with a previous history of gout, alcohol abuse, anaemia, neoplasm, chronic glomerulonephritis, abnormal liver function, bibulosity, acute complications of diabetes, severe cardiovascular, and cerebrovascular diseases were excluded. Patients taking medications for lowering or affecting UA levels in the last month (e.g., allopurinol, benzbromarone, aspirin, diuretics, losartan, pyrazinamide, cyclosporine, and azathioprine) and those taking SGLT2 inhibitors were also excluded.

### Measurements

General information, including sex, age, body weight, height, diabetic duration, medication history, and history of previous diseases, was recorded. Body mass index was calculated as body weight divided by height squared, and body surface area (BSA) was calculated using the Stevenson formula (BSA = 0.0061 × height + 0.0128 × weight – 0.1529)[12]. Current smoking was defined as continuous smoking ≥ 1 cigarette per day for more than 6 months. Hypertension was defined as systolic blood pressure ≥ 140 mmHg and/or diastolic blood pressure ≥ 90 mmHg, having hypertension in the past, or currently using antihypertension medications. For biochemical data, all blood samples were drawn from the participants after at least a 12-h fast. The haemoglobin A1c (HbA1c) level was measured using the Tosoh HLC-723G8 HbA1c analyser. Routine blood tests included total cholesterol (TC), triglyceride, high-density lipoprotein cholesterol, low-density lipoprotein cholesterol (LDL-C), SUA, alanine aminotransferase, aspartate transaminase, and serum creatinine (data not shown) and were performed using the Beckman AU5800 automatic biochemical analyser. We collected 24-h urine specimens for 2 consecutive days after stable glycaemic control was achieved, and the mean values were adopted. The 24-h UEUA and 24-h UEGL were measured using the Roche Cobas8000/c701 biochemical analyser.

Hyperuricaemia was defined as SUA levels > 416 μmol/L (1 mg/dl = 59.5 μmol/L) in men and 357 μmol/L in women [2, 13]. UEUA and CLUA were corrected by BSA. UEUA was calculated as the 24-h urinary uric acid (24-h UUA) divided by BSA. Meanwhile, CLUA was calculated using the Eq. (24-h UUA) × (urine volume per minute)/SUA/BSA. eGFR was estimated according to the equation developed by the Chronic Kidney Disease Epidemiology Collaboration [14].

### Statistical analysis

Given that all the continuous variables were skewed, data analysis were conducted after natural logarithmic transformation. Descriptive data were presented as geometric means (95% confidence intervals) for continuous variables or as percentages (%) for categorical variables. For the analysis of participant characteristics, the differences between sexes were tested using analysis of covariance for continuous variables and *chi*-square test for categorical variables.

Analysis of covariance was applied to compare the indicators of UA metabolism (including SUA, UEUA, and CLUA) across the UEGL quartile groups, with adjustment for age, sex, body mass index, diabetes mellitus duration, smoking, hypertension, HbA1c, TC, triglyceride, high-density lipoprotein cholesterol, and eGFR. Furthermore, multivariable linear regression models with UEGL as the independent variable and SUA, UEUA, and CLUA as the dependent variables were used to assess their relationships, with adjustment for the above covariates. *β* coefficients and 95% confidence intervals were reported. Finally, multivariate logistic regression was performed to evaluate the association between hyperuricaemia and quartiles of UEGL with adjustment for covariates as described previously. Multiple comparisons across different groups were corrected using the Bonferroni method.

We tested for multicollinearity using the variance inflation factors before performing multivariate analysis. Because the covariates of TC and LDL-C were colinear (variance inflation factor for LDL-C > 10) in the present study, LDL-C was excluded in the multivariate models. All the variance inflation factors of the covariates included in the final multivariate models were < 2.93, indicating that no collinearity was acceptable. Moreover, we tested the interaction of sex with UEGL by including the interaction terms in the final linear regression and logistic models (sex × UEGL) [15]. All statistical analyses were performed using SAS version 9.4 (SAS Institute Inc., Gary, NC, USA). All tests were two-tailed, and *P* < 0.05 was considered statistically significant.

## Results

The mean patient age was 51.7 (range 50.8–52.7) years, and there were 398 (62.7%) and 237 (37.3%) male and female patients, respectively. The patient characteristics are detailed in Table 1. Of the 17 factors, 8 were significantly different between the male and female patients; these were age, body mass index, number of current smokers, TC, high-density lipoprotein cholesterol, LDL-C, SUA, and UEGL. Meanwhile, there was no significant difference in diabetes mellitus duration, rate of hypertension, rate of hyperuricaemia, HbA1c, triglyceride, eGFR, UEUA, CLUA, and urine volume between the sexes.

**Table 1:** Participant characteristics (*n* = 635)

Characteristics	Total	Males (*n* = 398, 62.7%)	Females (*n* = 237, 37.3%)	*P*^a^
Participants (*n*, %)				*-*
Age	51.7 (50.8–52.7)	50.3 (49.1–51.4)	54.3 (52.7–56.0)	< 0.0001
BMI (kg/m^2^)	26.4 (26.1–26.7)	26.7 (26.3–27.2)	25.9 (25.3–26.4)	0.01
DM duration (years)	5.8 (5.4–6.3)	5.7 (5.1–6.3)	6.1 (5.3–6.9)	0.49
Smoking (*n*, %)	216 (34.0)	197 (49.5)	19 (8.0)	< 0.0001
Hypertension (*n*, %)	388 (61.1)	245 (61.6)	143 (60.3)	0.76
Hyperuricaemia (*n*, %)	98 (15.4)	62 (15.6)	36 (15.2)	0.90
HbA1c (%)	8.5 (8.3–8.6)	8.4 (8.2–8.6)	8.6 (8.4–8.9)	0.13
TC (mmol/L)	5.00 (4.91–5.10)	4.87 (4.76–4.99)	5.22 (5.07–5.38)	< 0.001
TG (mmol/L)	1.81 (1.72–1.91)	1.88 (1.75–2.01)	1.70 (1.58–1.83)	0.08
HDL-C (mmol/L)	1.12 (1.10–1.14)	1.08 (1.05–1.10)	1.19 (1.16–1.23)	< 0.0001
LDL-C (mmol/L)	3.12 (3.05–3.20)	3.00 (2.92–3.09)	3.33 (3.21–3.46)	< 0.0001
eGFR (mL/min/1.73 m^2^)	104.52 (103.72–105.32)	105.03 (104.02–106.05)	103.66 (102.37–104.97)	0.10
SUA (μmol/L)	303.6 (296.9–310.4)	321.4 (312.5–330.6)	275.8 (266.8–285.1)	< 0.0001
UEUA (mg/24 h/1.73 m^2^)	312.07 (296.74–328.20)	316.42 (294.34–340.16)	304.89 (287.31–323.56)	0.48
CLUA (mL/min/1.73 m^2^)	4.25 (4.02–4.49)	4.07 (3.76–4.40)	4.57 (4.28–4.87)	0.08
UEGL (g/24 h)	2.48 (2.11–2.91)	3.91 (3.25–4.71)	1.15 (0.87–1.51)	< 0.0001
Urine volume (L/24 h)	2.2 (2.2–2.3)	2.2 (2.1–2.3)	2.3 (2.2–2.4)	0.08

Continuous and categorical variables are expressed as geometric means (95% confidence intervals) and as percentages, respectively

*BMI* body mass index, *DM* diabetes mellitus, *HbA1c* haemoglobin A1c, *TC* total cholesterol, *TG* triglyceride, *HDL-C* high-density lipoprotein cholesterol, *LDL-C* low-density lipoprotein cholesterol, *eGFR* estimated glomerular filtration rate, *SUA* serum uric acid, *UEUA* urinary excretion rate of uric acid, *CLUA* renal clearance of uric acid, *UEGL* urinary excretion rate of glucose

^a^Analysis of covariance or chi*-*square test

The comparison of UA metabolism indicators, including SUA, UEUA, and CLUA, across the different UEGL quartile groups are presented in Table 2. Patients in the higher quartile of UEGL tended to have lower SUA levels than those in the lower quartile. The adjusted geometric mean (95% CI) for SUA across the UEGL quartile (Q) groups was 324.8 (312.1, 338.0) for Q1, 298.1 (287.0, 309.7) for Q2, 290.4 (279.5, 301.7) for Q3, and 284.6 (273.2, 296.5) for Q4 (*P* for trend = 0.0001). With Q1 as the reference group, comparisons showed significant differences for each group (Q2, Q3, and Q4) (all *P* < 0.05). In contrast, participants in the higher quartile of UEGL were more likely to have higher CLUA (*P* for trend < 0.0001), and a similar trend was observed for UEUA. Although the *P* value for trend of UEUA was 0.26 (*P* > 0.05), the UEUA of Q4 was significantly higher compared with that of Q1 (*P* = 0.01).

**Table 2:** Adjusted geometric means of uric acid metabolism by quartiles of UEGL (*n* = 635)

Categories	UEGL category				*P* for trend ^a^
	Q1	Q2	Q3	Q4	
Range (g/24 h)	0.01–0.59	0.61–4.03	4.07–12.43	12.51–57.08	–
No. of participants	159	159	158	159	–
SUA (μmol/L)	324.8 (312.1–338.0)^b^	298.1 (287.0–309.7)^c^	290.4 (279.5–301.7)^d^	284.6 (273.2–296.5)^d^	0.0001
UEUA (mg/24 h/1.73 m^2^)	280.22 (251.84–311.80)	315.35 (284.86–349.10)	305.70 (275.95–338.65)	362.41 (324.79–404.39)^e^	0.26
CLUA (mL/min/1.73 m^2^)	3.60 (3.31–3.91)	4.51 (4.17–4.88)^d^	4.57 (4.23–4.95)^d^	5.15 (4.73–5.60)^f^	< 0.0001

*UEGL* urinary excretion rate of glucose, *SUA* serum uric acid, *UEUA* urinary excretion rate of uric acid, *CLUA* renal clearance of uric acid, *Q1* quartile 1 (P0, P25), *Q2* quartile 2 (P25, P50), *Q3* quartile 3 (P50, P75), *Q4* quartile 4 (P75, P100)

^a^ Analysis of covariance; Adjusted for sex, age, body mass index, diabetes mellitus duration, smoking, hypertension, haemoglobin A1c, total cholesterol, triglyceride, high-density lipoprotein cholesterol, and estimated glomerular filtration rate. Test for trend based on variable containing median value for each quartile

^b^ Adjusted geometric mean (95% confidence interval) (all such values)

^c^ Significantly different from SUA of Q1, *P* = 0.02 (Bonferroni-corrected)

^d^ Significantly different from SUA, UEUA, and CLUA of Q1, respectively, *P* < 0.001 (Bonferroni-corrected)

^e^ Significantly different from UEUA of Q1, *P* = 0.01 (Bonferroni-corrected)

^f^ Significantly different from CLUA of Q1, *P* < 0.0001 (Bonferroni-corrected)

Table 3 shows the associations between UEGL and UA metabolism indicators in the multivariable linear regression model. UEGL was negatively correlated with SUA after adjusting for the above-mentioned covariates (*β* = − 0.023, 95% CI − 0.034 to − 0.013, *p* < 0.0001). However, UEGL was positively correlated with UEUA (*β* = 0.046, 95% CI 0.018–0.074, *p* = 0.001) and CLUA (*β* = 0.063, 95% CI 0.042–0.085, *p* < 0.0001) in the adjusted model.

**Table 3:** Influence of UEGL on uric acid metabolism indicators (*n* = 635)

Categories	*β* coefficient	95% CI	Standardized *β* coefficient	*P*^a^
SUA	− 0.023	− 0.034, − 0.013	− 0.169	< 0.0001
UEUA	0.046	0.018, 0.074	0.148	0.001
CLUA	0.063	0.042, 0.085	0.256	< 0.0001

Obtained by using multivariable linear regression models

*CI* confidence interval, *UEGL* urinary excretion rate of glucose, *SUA* serum uric acid, *UEUA* urinary excretion rate of uric acid, *CLUA* and renal clearance of uric acid

^a^Multivariable linear regression model with UEGL as the independent variable and uric acid metabolism indicators as the dependent variables. Adjusted for sex, age, body mass index, diabetes mellitus duration, smoking, hypertension, haemoglobin A1c, total cholesterol, triglyceride, high-density lipoprotein cholesterol, and estimated glomerular filtration rate

**Fig. 1:**
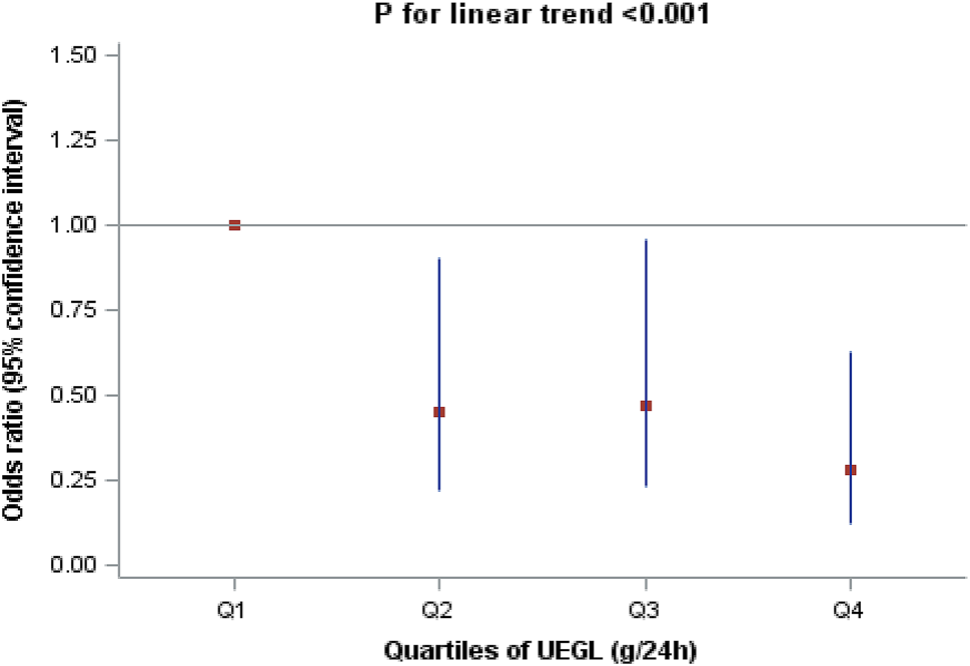
Adjusted odds ratios of hyperuricaemia across the UEGL quartiles* UEGL* urinary excretion rate of glucose, *Q1* quartile 1 (P0, P25), *Q2* quartile 2 (P25, P50), *Q3* quartile 3 (P50, P75), *Q4* quartile 4 (P75, P100). The odds ratio adjusted for sex, age, body mass index, diabetes mellitus duration, smoking, hypertension, haemoglobin A1c, total cholesterol, triglyceride, high-density lipoprotein cholesterol, and estimated glomerular filtration rate was calculated using the lowest UEGL Q1 subgroup as a reference

The results of multivariate logistic regression for the correlation of UEGL with hyperuricaemia are shown in Table 4 and Fig. 1. Consistent significant inverse associations were observed between quartiles of UEGL and hyperuricaemia. The fully adjusted odds ratios (OR) of hyperuricaemia across the UEGL quartile (Q) groups were 1.00 for Q1 (reference), 0.45 (0.22, 0.90) for Q2, 0.47 (0.23, 0.96) for Q3, and 0.28 (0.12, 0.63) for Q4 (*P* for trend < 0.001).

**Table 4:** Association between UEGL and HUA (*n* = 635)

Logistic regression models	UEGL category				*P* for trend ^a^
	Q1	Q2	Q3	Q4	
Range (g/24 h)	0.01–0.59	0.61–4.03	4.07–12.43	12.51–57.08	–
No. of subjects	159	159	158	159	–
No. of hyperuricaemia	37	22	23	16	–
Multivariable model ^b^	1.00 (reference)	0.45 (0.22, 0.90)^c, d^	0.47 (0.23, 0.96)^e^	0.28 (0.12, 0.63) ^f^	< 0.001

*UEGL* urinary excretion rate of glucose, *Q1* quartile 1 (P0, P25), *Q2* quartile 2 (P25, P50), *Q3* quartile 3 (P50, P75), *Q4* quartile 4 (P75, P100)

^a^Obtained by using logistic regression analysis. Test for trend based on variable including the median value for each quartile

^b^Adjusted for sex, age, body mass index, diabetes mellitus duration, smoking, hypertension, haemoglobin A1c, total cholesterol, triglyceride, high-density lipoprotein cholesterol, and estimated glomerular filtration rate

^c^Data are presented as odds ratio (OR) (95% confidence interval)

^d^Significantly different from UEGL of Q1, *P* = 0.03 (Bonferroni-corrected)

^e^Significantly different from UEGL of Q1, *P* = 0.04 (Bonferroni-corrected)

^f^Significantly different from UEGL of Q1, *P* = 0.003 (Bonferroni-corrected)

There were no statistically significant interactions between sex and UEGL (*P* for interaction = 0.15 in the multivariable linear model and *P* for interaction = 0.78 in the multivariable logistic model).

## Discussion

Few studies have evaluated on the relationship between UEGL and UA metabolism to date. In the present cross-sectional study, we demonstrated that high UEGL was strongly positively correlated with UEUA and CLUA. Moreover, UEGL was negatively associated with SUA level, and a consistently increased UEGL reduced the risk of hyperuricaemia in patients with T2D.

Epidemiological studies have shown that patients with diabetes were at a lower risk of developing gout than nondiabetes individuals [16], suggesting a potential association between UA metabolism and diabetes. Decreased SUA level may be among the mechanisms of the uricosuric effect of glycosuria, which occurs when the blood glucose level exceeds the maximum threshold for kidney reabsorption [4, 6]. Moreover, a study on uncomplicated type 1 diabetes suggested that glycosuria, rather than hyperglycaemia, increased uricosuria [17], and a similar result was observed in a Japanese clinical trial [10]. A recent study also demonstrated that the 2-h urinary glucose excretion was negatively correlated with SUA level in Chinese individuals newly diagnosed with diabetes [18]. Consistent with previous observations, the present study found that glycosuria was positively correlated with urinary excretion of UA, which was specifically manifested as high UEGL accompanied by high UEUA and CLUA. In contrast, patients in the higher quartile of UEGL tended to have lower SUA levels and lower risk of hyperuricaemia. In contrast, some studies showed that elevated SUA levels increased the risk of related chronic complications in obese adolescents with T2D and that diabetes patients are at higher risk of gout, with lower SUA associated with poor glycaemic control [3, 6]. A large retrospective cohort study reported that it is not diabetes itself but the comorbid conditions that increased the risk of gout [19]. In fact, the aetiologies of UA metabolism are complex. In addition, HbA1c and fasting glucose levels showed a bell-shaped relation with SUA levels [6]. This bell-shaped relationship raises an interesting implication of divergent risk of hyperuricaemia. Similarly, patients with T2D in the higher quintile of SUA were more likely to have lower HbA1c level in a large Italian observational study [20]. Notably, it is unclear whether there is a critical point for UEGL, that is, UA excretion will no longer increase when it reaches beyond a certain cut-off. Further larger studies are needed to clarify the hypothesis.

The specific mechanisms responsible for the uricosuric effect of glycosuria are yet to be clearly established. However, osmotic diuresis and/or a glomerular hyperfiltration state induced by glycosuria may play an important role [21]. In humans, UA is not further converted into soluble allantoin owing to the absence of uricase, which is readily eliminated in the urine. Approximately, 90% of UA is reabsorbed and secreted by the proximal tubules of the kidney, with the remaining part disposed by the gut [22]. Several anion transporters are involved in UA reabsorption, including urate transporter 1, glucose transporter 9 isoform 2, organic anion transporter 4, and organic anion transporter 10 [23–26]. Human glucose transporter 9 isoform 2, which is mostly located on the apical membrane of the proximal convoluted tubules, transport UA in exchange for urinary glucose at 10 mmol per litre [23]. Glucose transporter 9 isoform 2 as a facilitative glucose transporter is currently considered to play a major role in the uricosuric effect of glycosuria [10, 17, 24].

Observational studies displayed that elevated SUA levels were associated with various outcomes, including albuminuria, chronic kidney disease (CKD), cardiovascular events, and death [27–29], suggesting potential benefits of lowering UA. Indeed, several single-centre small sample trials demonstrated that urate reduction treatment with uric-acid-lowering drugs could slow the progression of CKD [30–33]. Also the systematic review and meta-analysis showed that uric acid-lowering therapy with allopurinol may delay the progression of CKD [34]. However, recent large randomized controlled trials showed no clinical benefits of serum urate reduction on kidney outcomes among patients with CKD and type 1 diabetes [35, 36]. And mendelian randomization studies suggested that SUA level was not a causal risk factor for renal complications in type 1 diabetes or in the general population [37, 38]. Moreover, the causality of SUA and increased risk of cardiometabolic traits is affected by multiple factors, potentially mediated through gout, hypertension and hypercholesterolemia [39]. These make it difficult to evaluate the clinical importance of lowering UA. what counts most is that it is currently unclear whether hyperuricemia plays a causative role in CKD progression or is merely a biomarker of decreased kidney function. In the future, more clinical trials will be needed to validate the association between SUA and CKD.

This study has some limitations. First, it was a single-centre cross-sectional study and did not prove causality. Second, to reduce the influence of renal insufficiency on uric acid excretion, only patients with basically normal renal function were included. Considering that some drugs can affect UA metabolism, we had to exclude a considerable number of individuals during screening, and this might have led to selection bias. Our participants only included those without taking drugs. However, this homogeneity can help reduce unmeasured confounding related to these drugs. Third, inconsistent glycaemic control may have an impact on urinary glucose excretion, although urine samples were collected after achieving stable glycaemic control. Despite these limitations, we believe that the present study remains valuable because it provides additional evidence to clarify the relationship between UEGL and UA metabolism in T2D.

In conclusion, our study confirmed that high UEGL was positively correlated with UEUA and CLUA. Moreover, high UEGL was inversely associated with SUA level, and consistently increased UEGL lowered the risk of hyperuricaemia in patients with T2D.

## Data Availability

The datasets generated for this study are available on request to the corresponding author.
